# Metabolic Side Effects of Risperidone in Pediatric Patients with Neurological Disorders: A Prospective Cohort Study

**DOI:** 10.3390/jcm13185565

**Published:** 2024-09-19

**Authors:** Jawza F. Alsabhan, Nouf Backer Al Backer, Fatimah M. Hassan, Awatif B. Albaker, Ghadeer Assiry

**Affiliations:** 1 Department of Clinical Pharmacy, College of Pharmacy, King Saud University, Riyadh 11322, Saudi Arabia; fatimah151194@gmail.com; 2 Department of Pediatrics, King Saud University Medical City, College of Medicine, King Saud University, Riyadh 11375, Saudi Arabiaghadeer.a.assiry@gmail.com (G.A.); 3 Department of Pharmacology and Toxicology, College of Pharmacy, King Saud University, Riyadh 11322, Saudi Arabia; abaker@ksu.edu.sa

**Keywords:** metabolic side effects, neurological disorders, pediatric patients, risperidone

## Abstract

**Background/Objectives:** Risperidone-related metabolic side effects in children have been primarily linked to variations between guideline-recommended and clinical treatment procedures. We explored the metabolic effects of risperidone administration in pediatric patients diagnosed with neurological disorders, thus evaluating its influence on metabolic indicators. **Methods:** This prospective cohort study gathered data from electronic health records, medical databases, and clinical reports. These data included patient demographics (e.g., age, sex, and body mass index) and information on risperidone use, including dosage, dosing frequency, and treatment duration. Additionally, laboratory tests were conducted at baseline and during treatment, along with other pertinent clinical variables. **Result:** A total of 52 eligible children (male; 73.1%) with neurological disorders treated with risperidone were included. The mean age was 13.4 ± 2.2 years. Risperidone was administered to 32.7% of patients for <2 years, 40.4% for 2–5 years, and 26.9% for 6–9 years, with a mean duration of 3.6 years. Most (53.8%) of the children experienced at least one metabolic side effect, with hyperlipidemia being the most common (34.6%). The median prolactin level increased slightly from 448.5 ng/mL at baseline to 479 ng/mL after 6–8 weeks. No significant associations were found between age, sex, duration of treatment, dosage form, dosing frequency, and hemoglobin A1c levels. **Conclusion:** Monitoring metabolic and anthropometric parameters in children receiving risperidone for neurological disorders is cardinal. Clinicians should consider individualized treatment plans, closely monitor metabolic markers, and address potential risks associated with long-term risperidone use in this vulnerable population.

## 1. Introduction

Neurological disorders in pediatric patients often present with challenging behavioral symptoms, necessitating pharmacological interventions [1]. These disorders encompass a diverse range of conditions affecting the central and peripheral nervous systems, presenting unique challenges for both patients and healthcare providers [2]. These disorders can manifest with a variety of symptoms, including developmental delays, seizures, impaired motor function, sensory deficits, and challenging behavioral symptoms. Understanding the intricate interplay between neurological dysfunction and behavior is crucial for effective management and improved quality of life in affected children [2]. Pharmacological management of neurological disorders in pediatric patients is a complex and nuanced process [3], with treatment strategies often aiming to alleviate symptoms, improve cognitive function, and enhance overall quality of life. It is important to note that the choice of medication, dosage, and duration of treatment should be individualized based on the specific neurological disorder, the severity of symptoms, and the overall health of the child.

Risperidone, an atypical antipsychotic, is commonly prescribed and approved by the United States Food and Drug Administration to treat symptoms of schizophrenia, manic episodes in patients with bipolar disorder, and irritability associated with autism spectrum disorder in pediatric patients [4]. It is often used off-label for a much broader range of mental disorders in young patients with attention deficit hyperactivity disorder [4].

However, concerns have been raised regarding the metabolic side effects associated with risperidone therapy. Recently, a systematic review of the literature from 2010 to 2020 on the monitoring of metabolic side effects in children and adolescents prescribed antipsychotic medication uncovered a gap between guideline-recommended procedures and clinical practice. Despite widespread concern about metabolic side effects, monitoring was inconsistent and infrequent, especially for biochemical parameters [5]. Another systematic review reported that the use of second-generation antipsychotics (SGAs), such as risperidone, requires appropriate monitoring procedures [6]. These treatment guidelines inform clinicians on how to deal with metabolic complications if they develop, and early detection and treatment of metabolic side effects would avoid disease progression and long-term complications. Therefore, regular monitoring of side effects and adjustments to treatment plans are essential.

One retrospective study conducted in 2011 on the risk of diabetes among pediatric patients who use SGAs or tricyclic antidepressants found that the risk of incident diabetes was significantly higher in the former group than in non-users of psychotropic medications, but was not significantly increased in comparison with those using antidepressants [7]. Another study examined the weight gain and metabolic consequences of risperidone in young children with autism spectrum disorder and concluded that rapid weight gain with risperidone treatment may promote a cascade of biochemical indices associated with insulin resistance and metabolic syndrome. Appetite, weight, waist circumference, liver function, blood lipids, and glucose levels should be monitored to avoid complications [8]. One prospective cohort study on the increased risk of obesity and metabolic dysregulation after 12 months of therapy with SGAs (risperidone or quetiapine) in children found an increased risk of developing obesity, elevated waist circumference, and dyslipidemia [9]. Regarding prolactin levels and associated side effects, one review reported that all antipsychotics, with the exception of clozapine, ziprasidone, and quetiapine, caused an increase in prolactin to levels higher than twice the upper limit of normal after 4–8 weeks of treatment [10]. In addition, other studies have reported increases in mean serum prolactin levels in children and adolescents receiving risperidone [11,12].

Understanding the underlying mechanisms is crucial for improving patient management and therapeutic strategies. Risperidone has been shown to affect the hypothalamic regulation of appetite and energy balance [13]. It increases appetite by blocking serotonin (5-HT2C) and histamine (H1) receptors, which are involved in hunger and satiety signals. The increase in caloric intake, combined with potential alterations in energy expenditure, leads to weight gain and changes in BMI [14]. Additionally, risperidone’s impact on leptin, an adipose tissue hormone involved in energy homeostasis, may contribute to its obesogenic effects [15]. Also, risperidone may alter the activity of lipoprotein lipase and other enzymes involved in lipid metabolism, leading to an imbalance in lipid profiles. Elevated triglycerides and altered cholesterol levels can increase the risk of cardiovascular complications [16]. Risperidone can affect glucose metabolism by interfering with insulin signaling pathways. It is known to cause insulin resistance through several mechanisms, including the inhibition of insulin receptor substrate (IRS) proteins and disruption of glucose uptake by peripheral tissues [17]. This can lead to elevated HbA1c levels and an increased risk of developing type 2 diabetes mellitus in susceptible individuals [18].

The objectives of this study were to assess the factors that contribute to the development of metabolic disorders during risperidone therapy, to assess the development of risperidone-related metabolic disorders, and to evaluate the metabolic side effects of risperidone therapy (diabetes, obesity, hyperprolactinemia, and dyslipidemia) in terms of pre- and post-therapy. We hypothesized that risperidone negatively impacts metabolic function in pediatric patients.

## 2. Materials and Methods

### 2.1. Study Design

This was a prospective cohort study with a predetermined follow-up period (6–8 weeks). This study followed the STROBE guidelines and was conducted at King Khalid University Hospital (KKUH), Riyadh, Saudi Arabia, from October 2023 to February 2024.

### 2.2. Participant Inclusion

Pediatric patients (adolescents) aged 10–18 years who were diagnosed with neurological disorders and prescribed risperidone were included in the study. The sample of participants was selected randomly from the clinic over 2 days weekly, without knowing any patient-specific information. Medical numbers were collected to order laboratory tests in the E-Sihi record system.

Patients with metabolic disorders, such as obesity, diabetes, hyperprolactinemia, and on other antipsychotic drugs, such as clozapine, olanzapine, and valproic acid were excluded from the study to reduce the risk of confounding variables that could skew the results. The enrolled patients were randomly selected from pediatric primary care clinics to ensure that the sample was representative of children receiving risperidone in a real-world clinical setting, making the findings relevant and applicable since ongoing monitoring is crucial for optimizing the benefits of risperidone while minimizing its risks. A well-structured monitoring plan ensures that potential side effects are detected early and managed effectively, supporting the overall well-being and treatment adherence of patients on risperidone. To ensure adequate statistical power, a priori power analysis for a *t*-test with two unpaired groups was conducted using G*Power 3.1, with a presumed effect size (d = 0.80) and α = 0.05 (two-tailed). Consequently, a total sample size of 52 participants was required to attain high statistical power (i.e., 0.80).

### 2.3. Data Collection

The first step in the participant recruitment process was to review the medical charts of patients. Subsequently, the demographic information of the patients, including diagnosis, sex, and age, was collected. We then added all the information about risperidone to the data collection sheet, including the dose, dosage form, and duration of treatment. Complete baseline assessments of the participants’ metabolic parameters (first record) including hemoglobin A1c (HbA1c) levels, body mass index (BMI), prolactin levels, and lipid profiles (low-density lipoprotein (LDL), high-density lipoprotein (HDL), and triglycerides) were obtained using standardized measurement tools and procedures. Next, we assessed the current levels of metabolic parameters after 6–8 weeks of treatment (second record) to track changes over time. The 6–8 week period was selected based on its common use in clinical trials and studies. This timeframe is typically sufficient to observe early side effects and is practical for study design [19] to assess the initial impact. Finally, the variables were categorized based on the collected parameters. For further visualization and elucidation, the demographic data were categorized according to gender: male participants were labeled 1 and females 2. Additionally, the participants’ ages were divided into three groups based on their age range of between 10 and 18 years old, and the age results were classified based on the following age groups: group 1, 10–12; group 2, 13–15; group 3, 16–18. This was done to compare the metabolic side effects of risperidone between different age groups based on similar mental and physical maturity.

With regard to metabolic parameters, HbA1c levels were categorized according to the American Diabetes Association, with the normal group categorized as 1, the pre-diabetic group as 2 if HbA1c levels were 5.7% to 6.4%, and the diabetic group as 3 if HbA1c levels were >6.5% [20].

In addition, BMI was measured and categorized according to the World Health Organization as underweight if BMI was <18.5 kg/m^2^ (group 1), normal weight if BMI was 18.5–24.9 kg/m^2^ (group 2), overweight if BMI was ≥25.0 kg/m^2^ (group 3), and obese if BMI was ≥30.0 kg/m^2^ (group 4) [21]. In addition, prolactin level was investigated, and a participant was considered to have hyperprolactinemia if two consecutive measurements were greater than 500 mIU/L [10]. Further, serum concentrations of cholesterol, HDL, and LDL were measured. Dyslipidemia was diagnosed if at least one lipid parameter was high (cut-off levels of >200 mg/dL for total cholesterol, >130 mg/dL for LDL, and <35 mg/dL for HDL) and if at least two out of three consecutive measurements were above their cut-off level. The subjects were then classified into normal and dyslipidemia groups [22].

### 2.4. Ethical Considerations

Approval was obtained from the Institutional Review Board of KKUH on 08 October 2023 (Approval number: E-23-7981), and informed consent was obtained from parents or guardians of the children to participate in the study. Privacy and security of patient information were maintained through redacting any patient-specific information. All collected data were anonymized. Personal identifiers were replaced with unique codes, ensuring that individual participants could not be identified from the published data. All research staff were trained in data privacy and security protocols, emphasizing the importance of maintaining confidentiality and protecting participant data.

### 2.5. Statistical Analysis

Statistical analyses were performed using the Statistical Package for Social Science software (version 27.0; IBM SPSS Statistics for Windows, version 27.0. Aromonk, NY, USA: IBM Corp). Descriptive statistics covered quantitative variables, whereas comparative analyses utilized chi-square and *t*-tests to provide a robust evaluation of the gathered information. All statistical tests were two-tailed and statistical significance was set at *p* < 0.05 for all tests. Descriptive analysis was performed using frequency distribution and percentages for study variables, including children’s age, sex, and lipid profile levels, and their metabolic disorder prevalence was graphed. All quantitative measures (lipid profile, laboratory findings, and anthropometric measures) are presented as means with standard deviations or medians, with ranges according to the shape of the distribution. BMI assessment was based on the growth chart percentiles for age and sex. Cross-tabulation was used to show factors associated with metabolic disorders using Pearson’s chi-square test for significance and exact probability tests if there were small frequency distributions. An independent samples *t*-test was used to compare mean values.

## 3. Results

**Figure 1:**
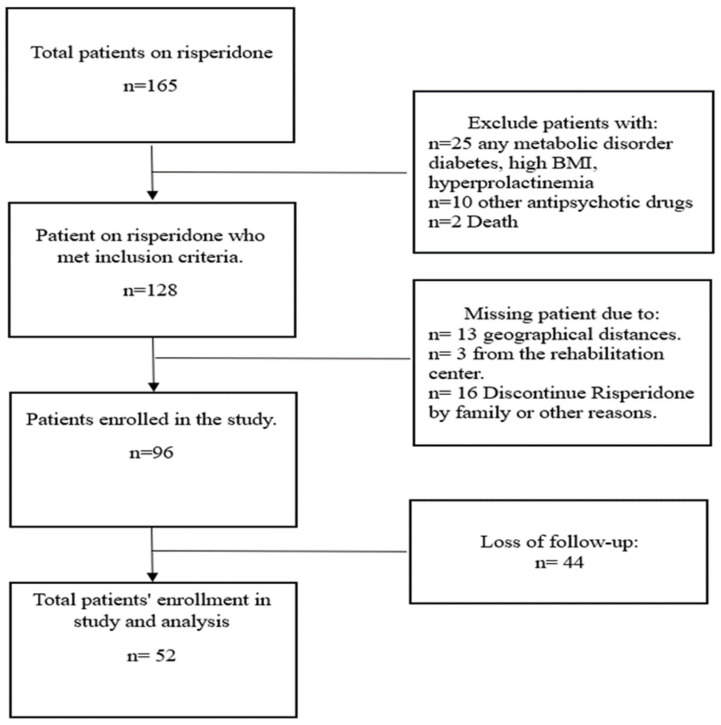
Enrollment flow chart of children with neurological disorders on risperidone.

A total of 52 eligible adolescent patients with neurological disorders who received risperidone therapy were included out of 165 screened patients (Figure 1). The majority of participants fell within the 13–15 age range (51.9%), followed by the 10–12 (30.8%), and the 16–18 (17.3%) age ranges. The mean age of the participants was 13.4 years, with a standard deviation of 2.2 years. In terms of sex, most participants were male (73.1%). The duration of risperidone use among the participants varied, with 32.7% using it for less than 2 years, 40.4% for 2–5 years, and 26.9% for 6–9 years. The mean duration of risperidone use was 3.6 years with a standard deviation of 2.5 years (Table 1).

**Table 1:** Demographic characteristics of study participants with neurological disorders receiving risperidone.

Personal Data	(n = 52)	%	Mean ± SD
Age (years)			
10–12	16	30.8%	13.4 ± 2.2
13–15	27	51.9%	
16–18	9	17.3%	
Gender			
Male	38	73.1%	
Female	14	26.9%	
Duration of treatment (years)			
<2	17	32.7%	3.6 ± 2.5
2–5	21	40.4%	
6–9	14	26.9%	

**Figure 2:**
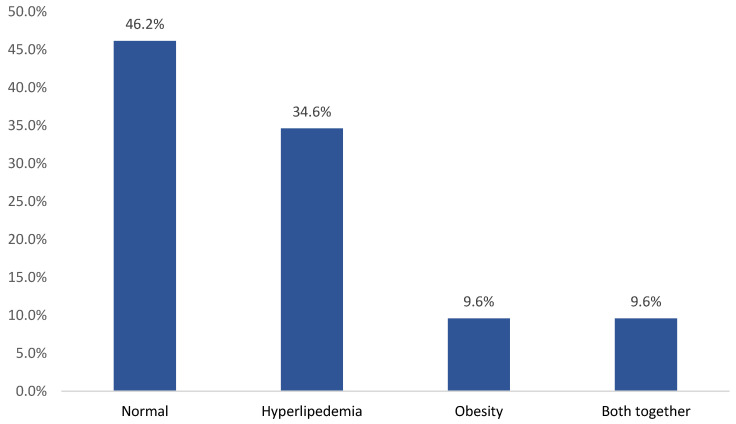
Prevalence of metabolic disorders among study children with neurological disorders receiving risperidone.

Regarding the prevalence of metabolic disorders, 28 (53.8%) children developed at least one metabolic side effect, including hyperlipidemia in 18 (34.6%), obesity in 5 (9.6%), and obesity and hyperlipidemia in 5 (9.6%) (Figure 2).

The route of risperidone administration, frequency of administration, and drug dosage among participants at baseline and after 6–8 weeks of risperidone use were investigated. At baseline, tablets were the preferred route of intake for 50.0% (n = 26) of the participants, followed by syrup in 48.1% (n = 25), and intramuscular (IM) injection in 1.9% (1). After 6–8 weeks, the preference for tablets increased slightly to 55.8% (n = 29), syrups decreased to 42.3% (n = 22), and IM injection remained at 1.9% (n = 1). In terms of the frequency of intake, most participants took their therapy daily, both at baseline (65.4%, n = 34) and after 6–8 weeks (69.2%, n = 36). The twice-daily intake decreased slightly from 32.7% (n = 17) at baseline to 28.8% (n = 15) after 6–8 weeks. The mean dosage of drugs increased slightly from 1.1 at baseline to 1.3 after 6–8 weeks, with standard deviations of 0.8 and 1.0, respectively.

**Figure 3:**
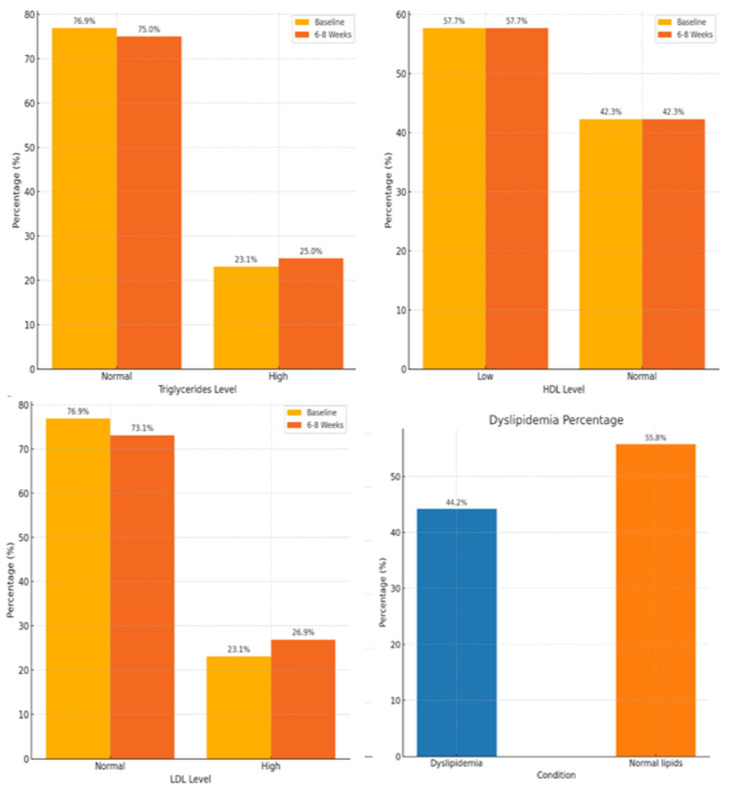
Changes in lipid profile and dyslipidemia prevalence over time.

Most participants had normal triglyceride levels both at baseline (76.9%, n = 40) and after 6–8 weeks (75.0%, n = 39). A slight increase in mean triglyceride levels was observed from 1.18 mmol/L at baseline to 1.26 mmol/L after 6–8 weeks. Approximately 57.7% (n = 30) of the participants had low HDL levels, whereas 42.3% (n = 22) had normal levels at baseline and after 6–8 weeks. The mean HDL levels showed a slight decrease from 1.51 mmol/L to 1.48 mmol/L during the observation period. Most participants had normal LDL levels at baseline (76.9%, n = 40), which decreased slightly to 73.1% (n = 38) after 6–8 weeks. To this end, the proportion of participants with high LDL levels increased from 23.1% (n = 12) to 26.9% (n = 14). There was an increase in mean LDL levels from 2.05 mmol/L at baseline to 2.20 mmol/L after 6–8 weeks. Dyslipidemia was present in 44.2% (n = 23) of the participants at baseline, and 55.8% (n = 29) had normal lipid profiles. There were no specific data regarding changes in the prevalence of dyslipidemia after 6–8 weeks (Figure 3).

**Figure 4:**
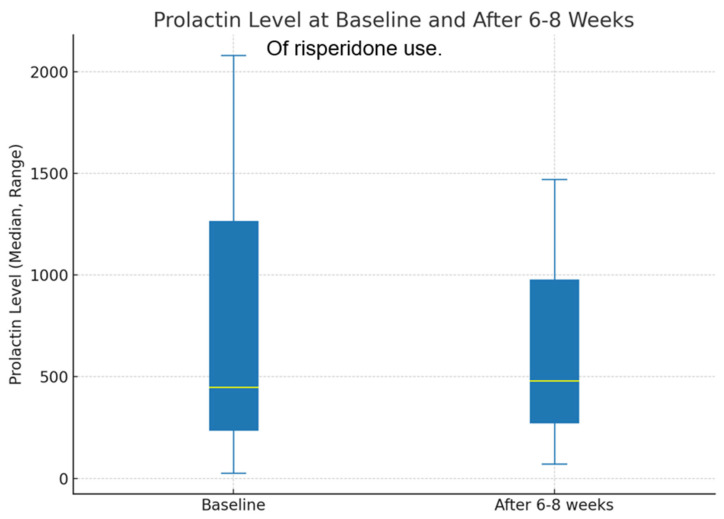
Prolactin levels at baseline and after 6-8 weeks of risperidone use.

Regarding the development of diabetes mellitus, 67.3% (n = 35) of the participants had normal HbA1c levels at baseline, whereas 32.7% (n = 17) were pre-diabetic. The mean HbA1c level was 5.4%, with a standard deviation of 0.4%. After 6–8 weeks, the percentage of participants with normal HbA1c levels decreased slightly to 57.7% (n = 30), whereas that of those who were pre-diabetic increased to 40.4% (n = 21). Additionally, 1.9% (n = 1) of the participants were diagnosed with diabetes. The mean HbA1c level increased to 5.6% after 6–8 weeks, with a standard deviation of 0.4%. At baseline, the median prolactin level was 448.5 ng/mL, ranging from 28.3 to 2081 ng/mL. After 6–8 weeks, the median prolactin level slightly increased to 479 ng/mL, with a range of 71.6 to 1472 ng/mL (Table 2 and Figure 4). The pairwise comparison showed that the median level appears slightly higher than the baseline but still close to 750 ng/mL.

**Figure 5:**
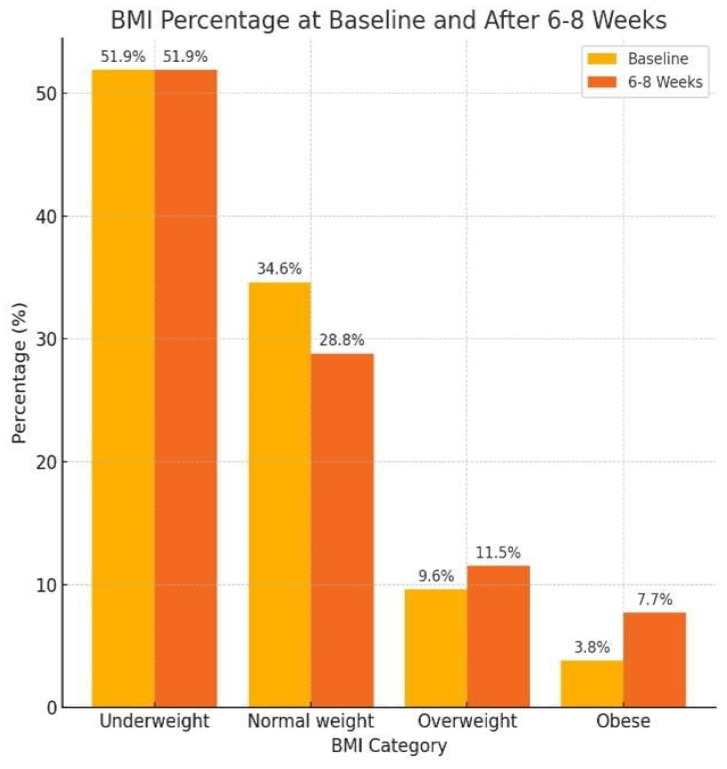
BMI percentage at baseline and after 6–8 weeks of risperidone use.

Regarding the performed anthropometric measurements (Figure 5), five (9.6%) children were overweight and two (3.8%) were obese at baseline. After 6–8 weeks, six (11.5%) became overweight and four (7.7%) obese. The mean body weight was 47.4 ± 17.4 kg, and the mean height was 155.1 ± 11.0 cm.

Table 3 below summarizes the factors associated with metabolic disorders in the study population. A total of 70.6% of patients who were administered risperidone for less than two years had more metabolic disorders than those who received therapy for 6–8 years (35.7%) (*p* = 0.049). In addition, the mean drug dose was significantly higher among cases with metabolic disorders than those without (1.31 ± 1.1 vs. 0.80 ± 0.4, respectively; *p* = 0.034).

Table 4 does not show any significant differences in dyslipidemia based on age, sex, treatment duration (years), dosage form, or dosing frequency. However, certain trends were observed; dyslipidemia was more common in younger patients aged 10–12 years (62.5%) than in older adolescents aged 16–18 years (44.4%), although the difference was not statistically significant (*p* = 0.177). Male and female patients had similar rates of dyslipidemia (44.7% vs. 42.9%, respectively, *p* = 0.905). Patients with a shorter duration of risperidone use (<2 years) had slightly higher rates of dyslipidemia (47.1%) than those with a longer duration (28.6% at 6–8 years), however, this difference was not statistically significant (*p* = 0.366). Regarding medication factors, patients taking tablets had lower rates of dyslipidemia (38.5%) than those taking syrups (48.0%) or receiving an injection (100%), however, these differences were not statistically significant (*p* = 0.416). Overall, although some trends were evident in relation to developing dyslipidemia, no factors were found to be significantly associated with the condition.

The association between HbA1c levels and age, sex, duration of therapy, dosage form, and dosing frequency, categorized as normal, pre-diabetic, and diabetic showed no significant association (*p* = 0.584) between age and HbA1c levels. However, participants aged 16–18 years showed a higher percentage of normal HbA1c levels than the younger age groups. Sex (*p* = 0.172) was not significantly associated with HbA1c levels. However, females tended to have a higher percentage of normal HbA1c levels than males. The duration of treatment (*p* = 0.540) was not significantly associated with HbA1c levels. There was no significant association (*p* = 0.290) between dosage form and HbA1c levels. However, participants receiving IM therapy or syrups tended to have a higher percentage of normal HbA1c levels than those taking tablets. Additionally, the frequency of intake (*p* = 0.514) was not significantly associated with HbA1c levels.

Table 5 shows statistically significant associations of participant BMI with age, dosage form, and dosing frequency (*p* = 0.001). Younger participants (10–12 years) were more likely to be underweight, whereas older participants (16–18 years) were more likely to be overweight or obese. Participants receiving IM risperidone were predominantly obese, whereas those taking syrups or tablets had varying BMI distributions. Participants taking risperidone daily or twice daily had higher rates of being overweight and obese than those taking risperidone less frequently. Sex (*p* = 0.369) did not demonstrate a significant association with BMI, although there was a trend of females being underweight compared to males. The treatment duration (*p* = 0.498) did not show a significant association with BMI.

## 4. Discussion

The findings of this study shed light on the various aspects of risperidone use in children with neurological disorders. Most participants were adolescents aged between 13 and 15 years old, with a higher prevalence of males. The duration of risperidone use varied, with a substantial proportion of participants using it for 2–5 years. This suggests a considerable reliance on risperidone for the management of neurological conditions in this population [19]. Significant metabolic effects associated with risperidone use were observed, including changes in HbA1c levels and lipid profiles. While most participants maintained normal triglyceride and LDL levels, there were fluctuations in HDL levels and a significant proportion developed dyslipidemia. Additionally, a notable percentage of participants experienced changes in HbA1c levels, with more participants shifting from normal to prediabetic levels after 6–8 weeks of observation. The observed changes in HbA1c levels and lipid profiles highlight critical concerns about the long-term metabolic impact of risperidone. The study’s findings of fluctuations in HDL levels and a notable shift in HbA1c from normal to prediabetic levels underscore the importance of proactive metabolic monitoring. These results are consistent with previous research indicating the potential for risperidone to induce metabolic dysregulation [16]. However, none of the other investigated factors including age, sex, treatment duration, dosage form, and dosing frequency showed significant associations with HbA1c levels. Nevertheless, older individuals and females showed a higher percentage of normal HbA1c levels, and participants on injection therapy and those administering syrups had higher percentages of normal HbA1c levels than those taking tablets. Further investigation is required to better understand these trends.

Additionally, the study highlighted significant shifts in BMI among participants, with a considerable proportion being underweight at baseline and presenting a slight increase in mean BMI after 6–8 weeks. The prevalence of obesity and being overweight also increased slightly during the study period. Several studies have suggested that risperidone increases body weight [23,24]. Additionally, weight gain from risperidone therapy is a significant factor that leads to non-compliance among children and adolescents. Therefore, it is imperative to assess and address weight gain as an outcome of risperidone treatment [25].

Prolactin levels also increased slightly from baseline to after 6–8 weeks. This finding suggests potential changes in hormonal profiles over the observed period. Clinical findings showed that risperidone elevates prolactin levels, leading to lactation dysfunction, which is a notable concern even in pediatric patients, as it can affect overall endocrine function, cause irregular menstrual cycles that impact reproductive health and overall quality of life, and reduce fertility, consequently affecting overall quality of life [26].

A significant association was observed between treatment duration and metabolic disorders. Participants using risperidone for a shorter duration (<2 years) and those receiving higher doses were more likely to experience metabolic complications. For more explanation, metabolic side effects of risperidone, such as weight gain and changes in glucose and lipid metabolism, are often more pronounced in the initial phases of treatment. New users might experience rapid metabolic changes as their bodies adjust to the medication. Over longer periods, patients and their healthcare providers may implement lifestyle modifications, such as dietary changes and increased physical activity, which can help mitigate some of the adverse metabolic effects. Some patients may develop a degree of metabolic tolerance over time, meaning that the initial sharp increases in metabolic parameters might stabilize or improve as the body adapts to the medication.

Age was significantly associated with changes in BMI, with younger participants being more likely to be underweight, and older ones being overweight and obese. In addition, the dosing frequency was associated with higher BMI values, emphasizing the importance of dosage management.

## 5. Conclusions

This study provided valuable insights into the use of risperidone in children with neurological disorders. These findings underscore the substantial reliance on risperidone for managing such conditions, particularly among adolescents, with the associated metabolic adverse effects being a significant concern. Metabolic side effects, including changes in lipid profiles and HbA1c levels, were evident among the participants, indicating the need for careful monitoring and management of these parameters during risperidone therapy. Practical recommendations for managing metabolic risperidone side effects are required. There should be a routine monitoring schedule implemented that includes regular measurements of HbA1c, lipid profiles (including HDL, LDL, and triglycerides), and BMI. Initial baseline assessments should be followed by periodic evaluations (every three to six months) to track changes and intervene early. Additionally, a regular check of prolactin levels, particularly if symptoms of hormonal imbalance are observed, should be carried out to address potential endocrine disruptions. Also, the dosage and treatment duration should be adjusted, based on individual patient responses. The lowest effective dose should be used for the shortest duration necessary to manage symptoms and minimize adverse effects. Moreover, age-specific factors should be considered when developing treatment plans, as younger patients and those with different dosing regimens may have varying risks for metabolic disturbances. Lifestyle and dietary interventions are recommended for controlling metabolic side effects from risperidone use. This recommendation can be done through collaboration between dietitians or nutritionists to provide personalized dietary guidance. Educate patients and their families about the importance of maintaining a healthy lifestyle to counteract potential weight gain and metabolic issues associated with risperidone.

The current study design did not include a placebo-controlled group, which limits our ability to distinguish the specific effects of risperidone from those displaying a natural progression of the neurological condition or subject to other external factors. Including a placebo group in future research would provide a clearer comparison and enhance the ability to attribute observed side effects directly to the medication. Another limitation of the study is the 6–8 week follow-up period used in this study, which may not be sufficient to capture all potential side effects of risperidone. While this duration is commonly used for initial assessments, it may miss delayed or cumulative adverse effects that develop over a longer period. Extending the follow-up period in future studies would help with identifying both short-term and long-term side effects more comprehensively.

Further research is crucial to better understand the mechanisms underlying risperidone’s metabolic side effects and their interactions with the pathophysiology of neurological disorders in pediatric patients. Long-term studies are needed to explore the chronic impact of risperidone and to refine strategies for managing its side effects. Additionally, research into alternative treatments or adjunctive therapies that minimize metabolic risks could enhance therapeutic options for this population. Also, there is a need for future studies that track risperidone levels in the blood and evaluate clinical assessments of safety and efficacy.

## Figures and Tables

**Table 2:** Laboratory findings among study children with neurological disorders receiving risperidone.

Laboratory Findings	(n = 52)	%	Mean ± SD
**HbA1c**			
At baseline			
- Normal	35	67.3%	5.4 ± 0.4%
- Pre-diabetic	17	32.7%	
After 6–8 weeks			
- Normal	30	57.7%	
- Pre-diabetic	21	40.4%	5.6 ± 0.4%
- Diabetic	1	1.9%	

**Table 3:** Association of demographic and treatment factors with metabolic disorders among patients on risperidone.

Factors	Metabolic Disorder				*p*-Value
	No		Yes		
	(n = 52)	%	(n = 52)	%	
Age (years)					0.110
10–12	6	37.5%	10	62.5%	
13–15	16	59.3%	11	40.7%	
16–18	2	22.2%	7	77.8%	
Gender					0.736
Male	17	44.7%	21	55.3%	
Female	7	50.0%	7	50.0%	
Treatment duration (years)					0.049 *
<2	5	29.4%	12	70.6%	
2–5	10	47.6%	11	52.4%	
6–9	9	64.3%	5	35.7%	
Dosage form					0.640 ^
IM injection	0	0.0%	1	100.0%	
Syrup	12	48.0%	13	52.0%	
Tablet	12	46.2%	14	53.8%	
Dosing frequency					0.418 ^
Daily	18	52.9%	16	47.1%	
Twice Daily	6	35.3%	11	64.7%	
Every 2 weeks	0	0.0%	1	100.0%	
Dose					
Mean ± SD	0.80 ± 0.4		1.31 ± 1.1		0.034 *#

P: Pearson X2 test; ^: exact probability test; #: independent *t*-test; * *p* < 0.05 (significant).

**Table 4:** Factors associated with developing dyslipidemia in the study population.

Factors	Dyslipidemia				*p*-Value
	Dyslipidemia		Normal Lipid Levels		
	(n = 52)	%	(n = 52)	%	
Age (years)					0.177
10–12	10	62.5%	6	37.5%	
13–15	9	33.3%	18	66.7%	
16–18	4	44.4%	5	55.6%	
Gender					0.905
Male	17	44.7%	21	55.3%	
Female	6	42.9%	8	57.1%	
Treatment duration (years)					0.366
<2	8	47.1%	9	52.9%	
2–5	11	52.4%	10	47.6%	
6–9	4	28.6%	10	71.4%	
Dosage form					0.416 ^
IM injection	1	100.0%	0	0.0%	
Syrup	12	48.0%	13	52.0%	
Tablet	10	38.5%	16	61.5%	
Dosing frequency					0.291 ^
Daily	14	41.2%	20	58.8%	
Twice Daily	8	47.1%	9	52.9%	
Every 2 weeks	1	100.0%	0	0.0%	

P: Pearson X2 test; ^: exact probability test.

**Table 5:** Factors associated with body mass index in the study population.

Factors	BMI								*p*-Value
	Underweight		Normal Weight		Overweight		Obese		
	(n = 52)	%	(n = 52)	%	(n = 52)	%	(n = 52)	%	
Age (years)									0.001 *
10–12	14	87.5%	2	12.5%	0	0.0%	0	0.0%	
13–15	12	44.4%	14	51.9%	1	3.7%	0	0.0%	
16–18	1	11.1%	2	22.2%	4	44.4%	2	22.2%	
Gender									0.369
Male	18	47.4%	14	36.8%	5	13.2%	1	2.6%	
Female	9	64.3%	4	28.6%	0	0.0%	1	7.1%	
Treatment duration (years)									0.498
<2	8	47.1%	5	29.4%	3	17.6%	1	5.9%	
2–5	13	61.9%	7	33.3%	0	0.0%	1	4.8%	
6–9	6	42.9%	6	42.9%	2	14.3%	0	0.0%	
Dosage form									0.001 *
IM injection	0	0.0%	0	0.0%	0	0.0%	1	100.0%	
Syrup	13	52.0%	9	36.0%	3	12.0%	0	0.0%	
Tablet	14	53.8%	9	34.6%	2	7.7%	1	3.8%	
Dosing frequency									0.001 *
Daily	19	55.9%	12	35.3%	3	8.8%	0	0.0%	
Twice Daily	8	47.1%	6	35.3%	2	11.8%	1	5.9%	
Every 2 weeks	0	0.0%	0	0.0%	0	0.0%	1	100.0%	

P: Exact probability test; * *p* < 0.05 (significant).

## Data Availability

The data presented in this study are available upon request from the corresponding author.
